# Silent Messengers: The Role of Extracellular Vesicle-Associated miRNAs in the Non-Invasive Profiling of Hepatocellular Carcinoma

**DOI:** 10.3390/biomedicines14061318

**Published:** 2026-06-10

**Authors:** Roxana-Luiza Caragut, Daniela Matei, Horia Stefanescu, Nadim Al Hajjar, Vasile Sandru, Ioana Berindan-Neagoe, Cristina Alexandra Ciocan, Laura Ancuta Pop, Zeno Sparchez

**Affiliations:** 1 “Iuliu Hațieganu” University of Medicine and Pharmacy, 400347 Cluj-Napoca, Romania; roxanacaragut@gmail.com; 2 Department of Gastroenterology, “Iuliu Hațieganu” University of Medicine and Pharmacy, 400347 Cluj-Napoca, Romania; horia.stefanescu@irgh.ro (H.S.); zsparchez@gmail.com (Z.S.); 3 Regional Institute of Gastroenterology and Hepatology, 400394 Cluj-Napoca, Romania; 4 Department of Surgery–Surgery III, “Iuliu Hațieganu” University of Medicine and Pharmacy, 400347 Cluj-Napoca, Romania; 5 Department of Gastroenterology, “Carol Davila” University of Medicine and Pharmacy, 050474 Bucharest, Romania; drsandruvasile@gmail.com; 6 Department of Gastroenterology, Clinical Emergency Hospital “Prof. Dr. Agrippa Ionescu”, 011356 Bucharest, Romania; 7 Department of Genomics, “Iuliu Hațieganu” University of Medicine and Pharmacy, 400347 Cluj-Napoca, Romania; ioana.neagoe@umfcluj.ro (I.B.-N.); cristina.ciocan@umfcluj.ro (C.A.C.); laura.pop@umfcluj.ro (L.A.P.)

**Keywords:** hepatocellular carcinoma, extracellular vesicles, extracellular vesicles microRNAs, liquid biopsy, non-invasive biomarkers, early diagnosis, prognostic biomarkers

## Abstract

Hepatocellular carcinoma (HCC) remains a major global health burden, characterized by late diagnosis, limited therapeutic options, and high mortality rates. Conventional diagnostic tools such as serum α-fetoprotein testing and imaging lack sufficient sensitivity for early detection. In recent years, liquid biopsy has emerged as a minimally invasive approach that enables real-time molecular profiling of tumors through the analysis of circulating biomarkers such as nucleic acids, proteins, and extracellular vesicles. Recent advances have underscored exosomes—nano-sized extracellular vesicles (EVs) secreted by nearly all cell types—as pivotal mediators of intercellular communication and dynamic carriers of tumor-derived molecular information, offering exciting prospects for early cancer detection and personalized therapy. In HCC, EV microRNAs (miRNAs) participate in multiple oncogenic processes, including proliferation, angiogenesis, epithelial–mesenchymal transition, and immune modulation. Specific EV-associated miRNAs, such as miR-21, miR-122, miR-224, and miR-221, show distinctive expression profiles in HCC and correlate with tumor stage, metastasis, and patient prognosis. Moreover, panels of circulating EV-associated miRNAs demonstrate superior diagnostic accuracy compared with traditional biomarkers, underscoring their potential as non-invasive tools for early detection and disease monitoring. Their inherent stability in biofluids and resistance to enzymatic degradation further support their application in liquid biopsy approaches. Despite promising results, continued research is essential to validate EV-associated miRNA signatures and to integrate these “silent messengers” into routine clinical practice for precision management of hepatocellular carcinoma.

## 1. Introduction

Hepatocellular carcinoma (HCC) is the primary malignant neoplasm of hepatocytes and represents the most common form of primary liver cancer, accounting for approximately 75–85% of cases. It typically arises in the setting of chronic liver disease and cirrhosis, most often due to viral hepatitis, chronic alcohol use, or metabolic dysfunction–associated steatotic liver disease. HCC is characterized by a multistep hepatocarcinogenic process that involves chronic inflammation, hepatocyte necrosis and regeneration, genomic instability, and progressive malignant transformation [1].

Traditional biomarkers, such as serum alpha-fetoprotein (AFP), and imaging modalities have demonstrated suboptimal sensitivity and specificity for early HCC detection and risk stratification [2]. Furthermore, tissue biopsy is constrained by invasiveness, potential sampling error owing to intratumoral heterogeneity, and often limited accessibility in cirrhotic patients [3]. In this context, liquid biopsy, the analysis of tumor-derived biomarkers circulating in body fluids, has emerged as a promising minimally invasive tool that might complement existing diagnostic and prognostic paradigms in HCC. Circulating tumor DNA (ctDNA), circulating tumor cells (CTCs), extracellular vesicles (EVs), and other cell-free nucleic acids provide a dynamic window into tumor biology, clonal evolution, and treatment response [4].

**Data sources:**

A literature search was conducted in PubMed to identify studies on extracellular vesicle-associated microRNAs (EV-associated miRNAs) in hepatocellular carcinoma (HCC). The search included publications available up to January 2026 and used combinations of the following terms: “extracellular vesicles”, “EV-associated miRNAs”, “exosomes”, “microRNAs”, “hepatocellular carcinoma”, “liquid biopsy”, “non-invasive biomarkers”, “diagnostic biomarkers”, “prognostic biomarkers”, “HCC diagnosis”, and “HCC prognosis”.

Original research articles, review articles, and meta-analyses published in English were considered for inclusion. Studies were selected based on their relevance to the diagnostic and prognostic role of EV-associated miRNAs in HCC. Articles not directly related to HCC, extracellular vesicles, or miRNA-based biomarkers were excluded. Additional relevant publications were identified through manual screening of the reference lists of selected articles.

## 2. Molecular Foundations of Liquid Biopsy in Hepatocellular Carcinoma

Hepatocellular carcinoma arises through a multistep carcinogenic process driven by chronic inflammation, oxidative stress, telomere dysfunction, and the accumulation of genetic and epigenetic alterations. These molecular events shape the tumor’s biological behavior and determine the composition of circulating biomarkers detectable through liquid biopsy. HCC is characterized by recurrent somatic mutations and pathway disruptions. Among the most frequent genetic alterations are TERT promoter mutations, which occur in approximately 40–60% of HCC cases and contribute to telomere maintenance and cellular immortalization [5].

Alterations of tumor suppressor genes are also common: TP53 mutations are associated with genomic instability, aggressive tumor phenotypes, and poor prognosis; they are particularly enriched in aflatoxin-related and HBV-associated HCC. The Wnt/β-catenin signaling pathway is involved in tumorigenesis, therapy resistance, and progression. Mutations in CTNNB1 result in constitutive activation of β-catenin signaling, promoting hepatocyte proliferation, immune evasion, and metabolic reprogramming [6].

Other key genomic alterations involve chromatin remodeling genes (e.g., ARID1A, ARID2), oxidative stress pathways (NFE2L2, KEAP1), PI3K–AKT–mTOR signaling (PIK3CA, PTEN), and RAS–MAPK signaling (RAS, BRAF). These disruptions collectively remodel hepatocyte identity, promote malignant transformation, and influence patterns of biomarker release [7,8].

The overexpression of Doublecortin-like kinase 1 (DCLK1) is linked to tumor growth, cancer stem cell self-renewal, metastasis, tumoral microenvironment regulation, and epithelial-to-mesenchymal transition (EMT). Increased DCLK1 expression has been associated with poor prognosis in HCC. DCLK1 can be considered a biomarker for the diagnosis and monitoring of HCC progression. Studies have demonstrated that DCLK1 is targeted by miRNAs such as miR-144 and miR-200a, suppressing DCLK1 expression, cancer cell growth, and invasion. On the other hand, DCLK1 silencing upregulates miR-143 and miR-145, which are tumor suppressors. Studies have indicated that miR-1246 levels and elevated DCLK1 are associated with inflammation-driven tumor development, while downregulation of miR-206 has been associated with HCC progression, including the transition from cirrhosis to malignancy [9].

Cell-free DNA (cfDNA) consists of fragments of DNA circulating in the bloodstream and originating mainly from the apoptosis of hematopoietic cells. DNA originating from tumor cells may carry specific genetic alterations and can be used as highly specific biomarkers for detection. Cell-free nucleic acids (cfNAs) can enter the circulation passively, mainly via necrosis and apoptosis, but also through active secretion via EVs. A thorough understanding of the origin and biological properties of ctNAs is essential for their effective application as clinical biomarkers. Their distinct features are closely linked to the mechanisms of their release and the dynamics of their clearance from the body. While circulating tumor DNA (ctDNA) contains key genetic alterations associated with tumor development, circulating tumor RNA (ctRNA) provides insight into dynamic processes occurring within and between tumor cells [10].

Studies have shown that ctDNA can detect mutations such as those in the TERT promoter, CTNNB1, and TP53 genes. Additionally, hypermethylation of the SEPT9 and RASSF1A genes in ctDNA has demonstrated potential in differentiating patients with HCC from individuals with benign liver diseases and healthy controls.

CTC molecular profiling can identify key mutations and phenotypic features, particularly in cells expressing epithelial–mesenchymal transition (EMT) markers, thereby supporting early detection of HCC [11].

miRNAs are short non-coding RNAs (about 22 nucleotides) that modulate gene expression and are key regulators of cancer development and progression. Prior studies have demonstrated that circulating miRNAs play a key role in tumor development by regulating the expression of both oncogenes and tumor suppressor genes. They control gene expression at the post-transcriptional level by binding to specific messenger RNAs (mRNAs), leading to either mRNA degradation or translation inhibition. Consequently, miRNAs modulate various cellular signaling pathways that are essential for cell growth, motility, proliferation, and survival. Importantly, many miRNAs are selectively packaged into extracellular vesicles (EVs), which protect them from degradation and facilitate their transfer between cells. Through EV-mediated intercellular communication, miRNAs can modulate the tumor microenvironment, promote angiogenesis and metastasis, and contribute to immune regulation in hepatocellular carcinoma, highlighting their relevance as both functional mediators and potential liquid biopsy biomarkers [12,13].

EVs comprise a heterogeneous population of membrane-bound vesicles, including exosomes, which are involved in intercellular communication through the transfer of proteins, lipids, and nucleic acids, including miRNAs, between donor and recipient cells [14]. As malignant hepatocytes undergo continuous proliferation, clonal selection, and cell death, molecular fragments reflecting these oncogenic processes are shed into peripheral blood. These include nucleic acids, proteins, metabolites, and other tumor-associated components that together constitute the molecular basis of liquid biopsy. Unlike tissue biopsy, which captures only a spatially and temporally restricted snapshot of tumor biology, liquid biopsy integrates molecular signals derived from multiple tumor regions and lesions, offering a more comprehensive representation of intratumoral and intertumoral heterogeneity [15]. A review of several studies has demonstrated that EV microRNAs and long non-coding RNAs are abundant in the circulation of patients with hepatocellular carcinoma and exhibit significant diagnostic and prognostic value. For example, specific EV-associated miRNAs, including miR-122, miR-145, miR-125b, and miR-192, have been reported in several studies to be associated with HCC and have the potential to distinguish malignant disease from liver cirrhosis, thereby providing diagnostic information at early disease stages. Furthermore, TP53 mutations identified in EV DNA have been associated with unfavorable clinical outcomes, including reduced recurrence-free survival and overall poor prognosis. In addition to their diagnostic and prognostic relevance, certain EV biomarkers have been implicated in tumor invasion and metastatic progression. Notably, plasma EV-associated miRNAs, such as miR-18a, miR-20b, and miR-27a, have been proposed as biomarkers of metastatic potential and disease aggressiveness, underscoring the central role of EVs in HCC pathophysiology [16].

### 2.1. Mechanisms of Release of Tumor-Derived Biomarkers

#### 2.1.1. Release of Circulating Tumor DNA (ctDNA)

Apoptosis is a major source of cell-free DNA in both normal and malignant tissues. Necrosis also contributes significantly to ctDNA release in more advanced or rapidly growing HCCs. Cells undergoing necrosis release longer, less fragmented DNA. Areas of intratumoral hypoxia promote necrotic cell death, thereby increasing ctDNA levels. There is evidence that suggests that tumor cells may actively secrete DNA via EVs or other vesicles, providing a protected form of ctDNA with distinctive methylation or mutational signatures [17]. In HCC, chronic inflammation, hypoxia, and immune-mediated cytotoxicity increase hepatocyte turnover, leading to the fragmentation and release of DNA fragments into the bloodstream. Tumor-derived apoptotic cfDNA often carries hallmark mutations, copy number variations, and methylation patterns characteristic of HCC [18].

#### 2.1.2. Release of Circulating Tumor Cells (CTCs)

HCC is highly vascular, often arising in a cirrhotic liver with abnormal angiogenesis and sinusoidal capillarization. Tumor cells can breach endothelial barriers and enter the bloodstream via single-cell invasion, collective migration, and transendothelial migration facilitated by vascular endothelial growth factor (VEGF) and matrix metalloproteinases (MMPs). Epithelial-to-mesenchymal transition (EMT) induces tumor cells to undergo modifications that facilitate detachment from the primary tumor and acquire mesenchymal characteristics, thereby acquiring a more invasive and migratory phenotype. Paracrine signaling from stromal cells or hypoxia can stimulate transcription factors that drive EMT [19]. Non-EMT-mediated translocation is associated with loss of cell-to-cell adhesion. Microvascular invasion (MVI), a hallmark of aggressive HCC, directly increases CTC shedding. Regions with disrupted endothelium allow tumor cell clusters to detach and enter the bloodstream [20].

#### 2.1.3. Release of Extracellular Vesicles

EVs have diameters ranging from 30 to 150 nm and are released into the extracellular space following the fusion of multivesicular bodies (MVBs) (exocytosis). EVs contain cargo that reflects their cell of origin, offering a dynamic spatiotemporal signature that could be exploited as a cancer biomarker to track disease progression in real time. Studies have identified several Rab family GTPases, such as Rab27a and Rab27b, as central regulators of the EV secretion pathway, with roles in tumor progression, providing early evidence that elements of the EV secretion pathway may contribute to tumor biology. In addition, Rab35 also regulates EV secretion through interactions with GTPase-activating proteins of the TBC1 domain family (TBC1D10A–C) [21,22]. EV secretion can also be stimulated by additional factors, including alterations in membrane pH and ceramide accumulation. As previously discussed, EVs are released into the extracellular space following the fusion of multivesicular bodies with the plasma membrane. Regulation of the EV biogenesis pathways is multifactorial and highly sensitive to intracellular calcium levels [23,24].

EVs are released from cells through two principal pathways: ectosome formation (also known as shedding microvesicles) and exosome release. Microvesicles originate through outward budding of the plasma membrane, whereas exosomes are generated within multivesicular bodies (MVBs) and released following their fusion with the plasma membrane. EVs are generated through the endosomal pathway, with intraluminal vesicle formation mediated in part by the endosomal sorting complex required for transport (ESCRT). HCC cells, which exhibit high metabolic and secretory activity, release large quantities of EVs containing DNA, RNA, lipids, proteins, and metabolites. Hypoxia, inflammation, and fibrosis stimulate EV biogenesis [23]. Hypoxic HCC cells release EVs enriched in pro-angiogenic factors (e.g., VEGF and miR-210), thereby promoting tumor survival and metastasis. HCC-derived EVs suppress natural killer (NK) cell and T-cell function and remodel the extracellular matrix, thereby facilitating immune evasion and enhancing tumor survival and progression [25].

Extracellular vesicles are highly abundant in circulation (approximately 10^10^ EVs per mL of plasma), although only a small proportion originates directly from the tumor. Their lipid bilayer membrane protects diverse molecular cargo, including proteins, nucleic acids, lipids, and metabolites, which are valuable for biomarker discovery. As EVs are continuously secreted by viable cells, they play an active role throughout all stages of cancer, from early development to metastasis. In addition, EVs, together with other tumor-derived factors, are released into the circulation by the primary tumor and contribute to the formation of pre-metastatic niches at distant sites. Locally, tumor-derived EVs can be transferred to surrounding normal stromal cells, reprogramming them into an altered, tumor-supportive state and inducing additional release of secondary EVs. Systemically, these tumor-derived EVs enter the peripheral circulation and are transported via the bloodstream to distant target cells, where they similarly reprogram recipient cells and promote secondary EV secretion. Collectively, as mentioned before, this process contributes to tumor progression, metastasis, and angiogenesis [26].

#### 2.1.4. Release of Circulating RNAs (cfRNA, miRNAs, lncRNAs) and DNAs

RNA and DNA can be released as a result of cell death (apoptosis or necrosis), binding to proteins (argonaute complexes), LDL, HDL, nucleosomes, or leakage from damaged hepatocytes [27]. A substantial proportion of cell-free RNAs in HCC is protected within EVs, where they may reflect the transcriptional activity of the parental tumor cells [28].

cfDNA consists of DNA fragments circulating in the bloodstream, primarily derived from the apoptosis of hematopoietic cells. DNA released by tumor cells may contain specific alterations that serve as highly sensitive and specific markers for detection. Notably, compared to healthy individuals, cancer patients tend to exhibit elevated levels of cfDNA, as well as increased amounts of mRNA and non-coding RNA in their blood. cfNAs (cell-free nucleic acids) can enter the circulation either passively, mainly via apoptosis and necrosis, or actively via secretion from viable cells into EVs. Gaining insight into the origin and properties of ctNAs is essential for their effective use as biomarkers in clinical applications [10,29].

In the bloodstream, circulating miRNAs are present in three main forms: free (naked) miRNAs, miRNAs associated with proteins such as Argonaute2 or high-density lipoproteins (HDL), and miRNAs enclosed within EVs. Their stability differs significantly: naked miRNAs are highly vulnerable to RNase-mediated degradation, protein-bound miRNAs exhibit intermediate stability, and EV-associated miRNAs show the greatest stability due to protection by a lipid bilayer. Among these, EV-associated miRNAs are considered particularly promising candidates for liquid biopsy applications. In contrast to naked and protein-bound miRNAs, which are often passively released from apoptotic or necrotic cells, EV-associated miRNAs are actively secreted through regulated pathways [30].

#### 2.1.5. Release of Tumor-Derived Proteins and Metabolites

Proteins such as AFP, AFP-L3, and PIVKA-II are actively secreted by malignant hepatocytes into the circulation. In advanced HCC, tumor hypoxia and necrosis promote the release of intracellular proteins and metabolic byproducts. Additionally, metabolic reprogramming involving lipid metabolism, glycolysis, and amino acid utilization generates measurable circulating metabolites [31].

## 3. EVs in Hepatocellular Carcinoma: Biogenesis and Molecular Cargo

Exosome biogenesis is a highly regulated process that plays a fundamental role in intercellular communication. Exosomes, a subtype of EVs, originate from the endosomal system and are generated within multivesicular bodies (MVBs), specialized endosomal compartments containing intraluminal vesicles (ILVs). These ILVs encapsulate a diverse array of bioactive molecules, including lipids, proteins, and nucleic acids. Upon fusion of MVBs with the plasma membrane, ILVs are released into the extracellular space as exosomes. Once secreted, exosomes can be internalized by recipient cells through endocytosis, facilitating the transfer of their molecular cargo and thereby modulating target cell function and behavior [32].

EV cargo is enriched in molecules involved in membrane trafficking and fusion, including Rab GTP-binding proteins, flotillins, and annexins, together with factors required for endosome formation, such as tumor susceptibility gene 101 (Tsg101). Proteins linked to lipid microdomains, notably integrins and members of the tetraspanin family (CD81, CD9, CD82, CD63, and CD83), are also commonly identified in EVs. The molecular profile of EVs reflects both shared features across species and variability determined by the cellular source from which they originate. Highly conserved components commonly reported include heat shock proteins (Hsps), CD63, and additional tetraspanins. Additionally, frequently detected constituents relate to structural organization and metabolic activity, including β-actin, cofilin, glyceraldehyde-3-phosphate dehydrogenase, tubulins, myosin, and major histocompatibility complex (MHC) class I and II proteins. Vesicles of this type further transport modulators of intracellular communication pathways, including elements associated with the Notch pathway ligand Delta-like 4, Wnt/β-catenin signaling, and interleukins [33].

Initially, EVs were regarded as cellular waste-disposal vesicles, and accumulating evidence supports their role as an alternative mechanism for eliminating unwanted cellular components to maintain cellular homeostasis. Beyond this function, EVs are now recognized as key mediators of intercellular communication and are involved in a broad range of functions. Notably, cancer cell–derived EVs have been shown to modulate immune responses, promote angiogenesis and remodel the surrounding tissue microenvironment, thereby contributing to tumor progression. In particular, EVs play a critical role in establishing the pre-metastatic niche [34].

EVs carry a diverse range of cellular constituents, including proteins, lipids, and several classes of nucleic acids, such as DNA, RNA, and mRNA, as well as characteristic surface protein markers, particularly tetraspanins. Due to their endocytic origin, EVs are enriched in endosome-associated proteins, including Rab GTPases, annexins, soluble N-ethylmaleimide-sensitive factor attachment protein receptors (SNAREs), and tetraspanins such as CD63, CD82, CD81, CD37, and CD53. Their lipid bilayer protects the molecular cargo from enzymatic degradation, thereby contributing to its stability in biological fluids. In addition, EVs contain miRNAs associated with RNA-binding proteins (RBPs), such as high-density lipoproteins (HDL), low-density lipoproteins (LDL), nucleophosmin 1, and Argonaute-2 (Ago2), which facilitate RNA loading into EVs and support RNA transport and stability [35].

Many different cell populations release EVs, and the molecular cargo they transport has been shown to influence communication both between cells and within cellular networks across numerous biological contexts. According to data compiled in the ExoCarta database, which catalogs molecular constituents associated with EV function, analyses from 146 published studies have identified thousands of cargo molecules, including 4563 proteins, 764 microRNAs, and 1639 messenger RNAs. These reported components illustrate the extensive range of biological processes potentially affected by EV-mediated transfer and underscore the relevance of investigating their roles in oncological research [36].

It is important to note that, in terms of diagnosis, more than seventy miRNAs have been proposed as novel molecular biomarkers [37].

In hepatocellular carcinoma, EVs exhibit unique properties that present promising opportunities for therapeutic intervention. EVs can be engineered to encapsulate a wide range of therapeutic payloads, including small-molecule drugs, nucleic acids, and proteins, and can be selectively directed toward tumor cells. As a therapeutic delivery system, EVs offer potential advantages by reducing systemic exposure and toxicity associated with conventional chemotherapy while enhancing therapeutic efficacy. Targeting key upstream signaling pathways implicated in HCC progression, including the Wnt/β-catenin, MAPK, and PI3K/Akt pathways, which regulate cell proliferation and survival, may therefore inhibit tumor growth and metastasis. Furthermore, endogenous EVs can be modified to deliver miRNAs or siRNAs that specifically suppress oncogenic transcripts, representing an emerging and highly adaptable therapeutic strategy [38]. EV DNA also represents a novel therapeutic avenue in HCC, as EVs carry double-stranded DNA of both nuclear and mitochondrial origin. This property enables engineering EV DNA to transport gene-editing systems, such as CRISPR–Cas9, to selectively disrupt pathogenic genetic alterations. Such an approach could achieve highly specific targeting of cancer cells with minimal off-target effects on healthy tissues [39].

## 4. EV-Associated miRNAs in Hepatocellular Carcinoma Pathophysiology

EV-mediated signaling enables malignant cells to modify surrounding cellular populations, thereby shaping conditions that favor tumor expansion. Conversely, vesicles released by immune and stromal compartments may exert suppressive or supportive effects on tumor development, reflecting their capacity to either promote or limit carcinogenic processes. These extracellular vesicles transport diverse molecular cargo, encompassing proteins, messenger RNA, microRNA, and other regulatory non-coding RNA species. By transferring such components, they modulate intracellular signaling and play significant roles in therapeutic resistance, neovascularization, epithelial–mesenchymal transition, and metastatic dissemination [40,41]. Beyond their biomarker potential, EV-based cell-free therapeutic approaches offer several advantages over cell-based therapies, including easier storage, scalable production, and reduced safety concerns, highlighting their promise as emerging therapeutic strategies [42].

Accumulating evidence indicates that EVs contribute to tumor progression by promoting the formation of an immunosuppressive microenvironment through signaling interactions between tumor cells and surrounding stromal cells. In particular, EV-associated microRNAs released from malignant cells and adjacent stromal components have been shown to support metastasis formation and dissemination [43]. Evidence suggests that in HCC, reduced levels of *EV-associated miR-320a* in cancer-associated fibroblasts (CAFs) activate downstream ERK signaling in recipient cells, thereby promoting lung metastasis [44]. Additionally, *EV-associated miRNA-1247-3p* secreted by cancer-associated fibroblasts can also promote lung metastasis in HCC [45]. Adipocytes have also been reported to release EV-associated *miR-23a/b*, which can be transferred to tumor cells and subsequently enhance the proliferation and migratory capacity of HCC cells [46]. Similarly, macrophage-derived EVs enriched in *miR-92a-2-5p* have been shown to promote the invasive capacity of HCC cells [47]. Furthermore, EVs produced under acidic microenvironmental conditions have been reported to be enriched *in miR-21* and *miR-10b*, which may further stimulate tumor growth and metastatic progression. Collectively, these findings highlight the dual role of EV-associated miRNAs as mediators of HCC progression and as potential therapeutic targets and prognostic biomarkers [48].

## 5. Diagnostic Value of EV-Associated miRNAs in Hepatocellular Carcinoma Early Detection and Disease Stratification

Numerous studies have focused on characterizing tumor cell–derived EV components to identify reliable biomarkers and potential therapeutic targets. Among these components, microRNAs (miRs) have received particular attention. Increasing evidence indicates that the incorporation of specific miRNAs into EVs is a regulated and selective process influenced by the biological properties of the parent cells, rather than a passive or random event. In addition, circulating miRNAs have emerged as promising non-invasive biomarkers in patients with HCC [49].

A study published in 2024 analyzed a cohort comprising 88 patients with HCC and 179 non-HCC controls, including 49 healthy individuals, 62 patients with hepatitis, 54 with cirrhosis, and 14 with benign hepatic tumors. Next-generation sequencing (NGS) was used to compare miRNA expression profiles in fucosylated extracellular vesicles (Fu-EVs) between HCC patients and non-HCC controls. A total of 2278 known miRNAs were identified. Differential expression analysis revealed 112 miRNAs significantly altered between the HCC and non-HCC groups. Five miRNAs (*hsa-let-7a*, *miR-150*, *miR-21*, *miR-200a*, and *miR-125a*) were identified as biomarkers for HCC diagnosis. The five-miRNA panel showed strong diagnostic performance for HCC, with a sensitivity of 0.90 and specificity of 0.92 in a cohort of 194 patients diagnosed with HCC and 412 non-HCC controls, outperforming both AFP and des-gamma-carboxy prothrombin (DCP). Importantly, the model achieved recall rates of 85.7% for stage 0 and 90.8% for stage A HCC, detected 88.1% of AFP-negative cases, and effectively distinguished HCC from other cancer types, offering a rapid and non-invasive strategy for early HCC detection [50].

In a study conducted by Cho et al., the expression levels of six miRNAs in serum-derived EVs were assessed across multiple cohorts to evaluate their potential as diagnostic biomarkers for hepatocellular carcinoma. One of the cohorts consisted of 24 participants, including healthy controls, patients with chronic hepatitis (CH), liver cirrhosis (LC), and HCC. The expression patterns of the six selected serum EV-associated miRNAs (*miR-25-3p*, *miR-140-3p*, *miR-423-3p*, *miR-1269a*, *miR-4661-5p*, and *miR-4746-5p*) were subsequently analyzed. The results demonstrated that serum EV-associated *miR-25-3p*, *miR-4661-5p*, *miR-1269a*, and miR-4746-5p were significantly upregulated in patients with HCC compared with healthy controls and patients with CH and LC. Receiver operating characteristic (ROC) curve analysis revealed that four of the six miRNAs, *miR-25-3p*, *miR-4661-5p*, *miR-1269a*, and *miR-4746-5p*, achieved an area under the ROC curve (AUROC) greater than 0.8, demonstrating good discriminative ability for distinguishing patients with HCC from those with CH and LC. Serum EV-associated miR-4661-5p demonstrated the highest diagnostic performance for hepatocellular carcinoma, with an AUROC of 0.918. The corresponding AUROC values for the remaining serum EV-associated miRNAs were 0.758 for *miR-25-3p*, 0.844 for *miR-1269a*, and 0.687 for *miR-4746-5p*. In addition, in distinguishing HCC from chronic hepatitis and liver cirrhosis, serum EV-associated *miR-25-3p*, *miR-4661-5p*, and *miR-1269a* demonstrated superior diagnostic performance, with AUROC values of 0.690, 0.910, and 0.829, respectively, compared with serum AFP, which showed an AUROC of 0.597. Moreover, elevated expression of *miR-25-3p*, *miR-140-3p*, and *miR-423-3p* was significantly associated with reduced overall survival (OS). In contrast, increased levels of *miR-1269a*, *miR-4746-5p*, and *miR-4661-5p* were significantly correlated with shorter disease-free survival (DFS). The diagnostic performance of serum EV-associated miRNAs was further evaluated across different modified Union for International Cancer Control (mUICC) stages of hepatocellular carcinoma. For distinguishing mUICC stage I/II HCC from non-tumor controls, serum EV-associated *miR-1269a* (AUROC = 0.853) and *miR-4661-5p* (AUROC = 0.910) demonstrated superior diagnostic accuracy compared with serum AFP (AUROC = 0.597). When differentiating stage I/II HCC from patients with chronic hepatitis or liver cirrhosis, *miR-4661-5p* (AUROC = 0.910) and miR-1269a (AUROC = 0.583) again outperformed AFP (AUROC = 0.540). The authors further assessed the ability of these biomarkers to detect early-stage HCC (equivalent to mUICC stage I). Serum *EV-associated miR-25-3p* (AUROC = 0.812), *miR-1269a* (AUROC = 0.684), and *miR-4661-5p* (AUROC = 0.923) exhibited greater diagnostic performance than AFP (AUROC = 0.541). Subgroup analysis was subsequently conducted to determine whether these candidate miRNAs could distinguish early-stage HCC from high-risk individuals. In this setting, serum *EV-associated miR-1269a* (AUROC = 0.837) and *miR-4661-5p* (AUROC = 0.924) demonstrated improved diagnostic accuracy compared with AFP (AUROC = 0.604) among patients with CH or LC [51]. Overall, these findings indicate that serum EV-associated miRNAs exhibit strong diagnostic potential for hepatocellular carcinoma, with several candidates achieving good to excellent discriminative performance (AUROC > 0.8) in distinguishing HCC from chronic hepatitis and liver cirrhosis. Among them, *miR-4661-5p* consistently demonstrated the highest diagnostic accuracy, outperforming the conventional biomarker AFP across multiple comparisons, including early-stage disease. Notably, certain EV-associated miRNAs also showed enhanced sensitivity for early HCC detection and for differentiating high-risk populations, underscoring their potential clinical utility as non-invasive biomarkers for early diagnosis and risk stratification in HCC.

A study by Yang et al. found significantly reduced expression of *miR-26a*, *miR-29c*, *and miR-199a* in serum-derived EVs from patients with HCC compared with individuals with hepatic cirrhosis and healthy controls. Similarly, plasma levels of these miRNAs were markedly decreased in patients with HCC relative to the comparison groups. Notably, the differences in expression were more pronounced in EV samples than in plasma. Compared with plasma miRNAs and serum AFP, EV-associated miRNAs (*miR-26a*, *miR-29c*, and *miR-199a*) demonstrated significantly greater accuracy in differentiating HCC from both healthy controls and patients with liver cirrhosis. In addition, tissue-based analysis further supported their diagnostic value, with area under the curve (AUC) values of 0.8901 for *miR-26a*, 0.9171 for *miR-29c*, and 0.8501 for *miR-199a*. Overall, in this study, EV-associated miRs demonstrated superior diagnostic performance compared with plasma miRNAs and AFP. These miRNAs were subsequently integrated into a diagnostic panel based on the following model: logit (*p* = HCC) = 7.401 − 3.724 × miR-26a − 2.894 × miR-29c − 7.430 × miR-199a. This EV-associated miRNA panel demonstrated excellent diagnostic accuracy, achieving a sensitivity of 100%, specificity of 96%, and an AUC of 0.994 for differentiating HCC from healthy individuals and also differentiating HCC from hepatic cirrhosis with a sensitivity of 92%, a specificity of 90%, and an AUC of 0.965. The tissue-based miRNA panel was also evaluated. Although it showed good diagnostic performance in distinguishing HCC from healthy controls (sensitivity = 85%, specificity = 96%, AUC = 0.957), its accuracy was comparatively lower than that of the EV-associated miRNA panel [52].

Another study involving 72 HCC patients reported that serum EV-associated miRNAs outperformed their freely circulating plasma counterparts in differentiating HCC from HBV-infected or cirrhotic patients and concluded that the expression levels of *miR-26a*, *miR-21*, and *miR-29c* were significantly reduced in patients with HCC compared with cirrhotic and HBV groups. These miRNAs were identified as independent diagnostic biomarkers for HCC [53].

Another study demonstrated that *miR-720* is a candidate biomarker for HCC, based on its significant differential expression between tumor and adjacent non-tumor tissues. Serum-derived EV-associated *miR-720* levels were markedly elevated in patients with HCC compared with those with other liver diseases, demonstrating high diagnostic accuracy (AUC = 0.931). Notably, EV-associated *miR-720* exhibited superior performance in detecting small HCC lesions (<5 cm), achieving an AUC of 0.930, exceeding that of AFP (AUC = 0.802) and protein induced by vitamin K absence-II (PIVKA-II; AUC = 0.718). Although EV-associated miR-720 levels exhibited only a weak association with tumor size, the proportion of patients with elevated *miR-720* increased with advancing intrahepatic tumor stage [54].

According to Sun et al., *miR-101* and *miR-125b* were significantly downregulated in both tumor tissues and serum-derived EVs from patients with hepatocellular carcinoma. ROC curve analysis demonstrated that circulating *EV-associated miR-101* and *miR-125b* achieved an AUC of 0.894 (95% CI, 0.793–0.994) and 0.812 (95% CI, 0.675–0.950), respectively. Notably, the combined analysis of these two miRNAs further improved diagnostic performance, achieving an AUC of 0.953 for HCC detection [55].

Cho et al. evaluated the diagnostic capability of serum *EV-associated miR-10b-5p* for distinguishing early-stage HCC from individuals at high risk of developing HCC. The AUC for differentiating HCC (all stages) from the high-risk group was 0.925. For stage I and stage II HCC, the AUC was 0.941. Moreover, in distinguishing modified Union for International Cancer Control (mUICC) stage I HCC from high-risk patients, the AUC reached 0.935, with a sensitivity of 90.6% and a specificity of 78.3% at a cutoff value corresponding to a 1.8-fold change [56].

Chen et al. investigated the potential clinical value of serum *EV-associated miR-34a* for early HCC diagnosis. The AUC for serum *EV-associated miR-34a*, AFP, and their combined use was 0.664 ± 0.0499, 0.826 ± 0.0396, and 0.855 ± 0.0337, respectively. The sensitivity and specificity were 78.3% and 51.7% for serum *EV-associated miR-34a*, 61.7% and 98.3% for AFP, and 68.33% and 93.33% for the combined detection strategy. These findings suggest that integrating serum *EV-associated miR-34a* with AFP may enhance diagnostic accuracy and support its potential utility in the early clinical detection of HCC [57].

Jingwen et al. reported that serum-derived EV *hsa-miR-27a-3p* and *hsa-miR-493-3p* were identified as potential diagnostic biomarkers for HCC. Both miRNAs displayed significant expression differences and showed superior diagnostic performance compared with AFP alone. Notably, combining these two EV-associated miRNAs with AFP achieved the highest diagnostic accuracy, underscoring the incremental value of incorporating EV biomarkers into conventional screening approaches for early HCC detection. The integration of EV-associated miRNAs into AFP-based surveillance strategies may improve early diagnosis and risk stratification, thereby potentially enhancing clinical outcomes [58].

Wang et al. reported that EV levels of *miR-122*, *miR-96*, and *miR-21* exhibited superior diagnostic sensitivity and specificity compared with their plasma counterparts for HCC. Based on these findings, the authors performed multivariate logistic regression analysis incorporating the three miRNAs and constructed a combined miRNA panel for HCC diagnosis. The diagnostic performance of this panel was subsequently evaluated in differentiating HCC from both healthy controls and patients with liver cirrhosis. The combined EV-associated miRNA panel demonstrated strong discriminative ability, with an AUC of 0.924 for distinguishing HCC from liver cirrhosis (sensitivity 82%, specificity 92%) and an AUC of 0.996 for differentiating HCC from healthy controls (sensitivity 96%, specificity 98%) [59].

Sohn et al. observed significant differences in the expression profiles of ten serum-derived EV microRNAs among patients with chronic hepatitis B (CHB), liver cirrhosis (LC), and HCC. Compared with the CHB group, patients with HCC exhibited markedly elevated levels of *EV-associated miR-18a*, *miR-221*, *miR-222*, and *miR-224*. In contrast, EV-associated *miR-101*, *miR-106b*, *miR-122*, and *miR-195* were significantly reduced in the HCC group relative to CHB patients. No statistically significant differences were detected in the levels of *EV-associated miR-21* or *miR-93* between the two groups. When comparing HCC and LC, serum EV levels of *miR-18a*, *miR-221*, *miR-222*, and *miR-224* were significantly elevated in patients with HCC. In contrast, *EV-associated miR-101* was significantly reduced in the HCC group. No significant differences were observed between HCC and LC in the EV expression of *miR-21*, *miR-93*, *miR-106b*, *miR-122*, or *miR-195* [60].

In another study, 90 patients with HCC and 41 healthy volunteers without liver disease were enrolled. Plasma-derived EVs were isolated from all participants, and the expression levels of five candidate miRNAs—*miR-183-5p*, *miR-34a-5p*, *miR-148b-3p*, *miR-19a-3p*, and *miR-215-5p* were analyzed to evaluate their diagnostic potential. ROC curve analysis confirmed strong diagnostic performance of the five candidate miRNAs for distinguishing HCC patients from controls. The AUC values were high: 0.9576 for *miR-148b-3p*, 0.9224 for *miR-19a-3p*, 0.9233 for *miR-183-5p*, and 0.9115 for *miR-34a-5p*. Overall, sensitivity ranged from 84.44% to 94.44% and specificity from 82.93% to 95.12%, with Youden’s Index between 0.6737 and 0.7981. *miR-148b-3p* showed the highest sensitivity (94.44%), while *miR-215-5p* had the greatest specificity (95.12%). When stratified by TNM stage, the miRNAs maintained strong performance (a sensitivity between 80.65% and 95.45%, a specificity between 75.61% and 97.56%, and Youden’s Index between 0.5656 and 0.8743). *miR-148b-3p* and *miR-215-5p* performed best across TNM stages. Although these markers could not distinguish between specific stages or grades, they effectively differentiated HCC patients from healthy individuals [61].

Moreover, EV-associated miRNAs are linked with histopathological changes in NAFLD. In particular, several circulating miRNAs, including *miR-34a*, *miR-192*, *miR-122*, and *miR-200a*, were found to correlate strongly with liver fibrosis stages. Multivariate analyses further indicated that *miR-34a*, *miR-192*, and *miR-122* are independently associated with hepatic steatosis and fibrosis, whereas *miR-200a* shows a specific association with fibrosis. Among these, *miR-34a* demonstrated the strongest predictive value for fibrosis severity [62].

*EV-associated miR-182*, *miR-373*, and *miR-301a* have been implicated in HCC pathogenesis, although data on EV-associated miRNAs in NASH-related cirrhosis with HCC remain limited. Different studies showed that all three miRNAs were significantly upregulated in HCC patients in both serum and ascitic fluid. These results suggest that serum and ascitic fluid *EV-associated miR-182*, *miR-373*, and *miR-301a* may serve as potential biomarkers for NASH-related HCC [63].

The main findings from studies investigating the diagnostic value of EV-associated miRNAs in HCC are summarized in Table 1, including their expression patterns and diagnostic performance.

A study aimed to compare the diagnostic performance of AFP, EV-miR-19-3p, the combination of EV-miR-19-3p and AFP, as well as EV-associated miR-30d-5p, EV-associated miR-16-5p, EV-associated miR-451a, and EV-associated miR-223-3p in the detection of Non-B, non-C hepatocellular carcinoma (NBNC-HCC). The findings demonstrated, for the first time, that EV-miR-19-3p represents a promising biomarker for NBNC-HCC diagnosis, exhibiting high diagnostic accuracy, particularly in AFP-negative cases. Furthermore, the combined use of EV-miR-19-3p and AFP improved overall diagnostic performance. These results highlight the potential of EV-miR-19-3p as a sensitive biomarker for early HCC detection and as a complementary marker in AFP-negative HCC. The other evaluated miRNAs (EV-miR-30d-5p, EV-miR-16-5p, EV-miR-451a, and EV-miR-223-3p) showed only moderate diagnostic performance [64]. These results, summarized in Table 2, highlight the potential of EV-miR-19-3p as a sensitive biomarker for early HCC detection and as a complementary marker in AFP-negative HCC.

## 6. Prognostic and Predictive Significance of EV-Associated miRNAs Association with Disease Progression and Survival, Recurrence, and Treatment Response

EV microRNAs have also gained increasing attention as potential prognostic indicators in hepatocellular carcinoma, owing to their stability in circulation and their close association with tumor biology.

Liu et al. evaluated the prognostic significance of microvascular invasion (MVI) and *EV-associated miR-125b* in patients with HCC using ROC curve analysis to determine sensitivity, specificity, and AUC. EV-associated *miR-125b* demonstrated favorable predictive performance for postoperative outcomes, with an AUC of 0.739 for recurrence and 0.702 for overall survival following liver resection. Moreover, the combined assessment of *EV-associated miR-125b* and MVI improved prognostic discrimination, achieving AUC values of 0.807 for recurrence and 0.765 for survival [65].

Yang et al. demonstrated that EVs derived from highly metastatic HCC cells can confer metastatic potential to recipient tumor cells through the transfer of *miR-92a-3p*. It was demonstrated that *miR-92a-3p* promotes epithelial–mesenchymal transition and tumorigenesis by activating the Akt/Snail signaling pathway via selective suppression of the tumor suppressor PTEN. In addition, the transcription factors E2F1 and c-Myc were found to be upregulated and to directly drive *miR-92a-3p* expression in HCC, thereby enhancing metastatic capacity. Importantly, circulating *EV-associated miR-92a-3p* levels in plasma were positively correlated with metastatic status in patients with HCC. These findings align with a previous study identifying *miR-92a-3p* as a promoter of cancer progression and provide comprehensive evidence linking *EV-associated miR-92a-3p* to metastasis in HCC [66].

The aforementioned study conducted by Cho et al. further assessed the prognostic significance of serum EV-associated miRNAs in HCC. Kaplan–Meier survival analysis demonstrated that elevated serum *EV-associated miR-215-5p* expression was significantly associated with shorter disease-free survival compared with low expression levels. Moreover, serum *EV-associated miR-215-5p* expression progressively increased with advancing tumor stage and was significantly higher in patients with vascular invasion than in those without. Serum *EV-associated miR-10b-5p* was not significantly associated with tumor stage or vascular invasion [56].

Yokota et al. investigated the clinical relevance of serum *EV-associated miR-638*, *miR-663a*, *miR-3648*, and *miR-4258* in a cohort of 54 patients with HCC who underwent hepatectomy. Preoperative serum EVs were analyzed, and patients were stratified into high- and low-expression groups according to the median value of each miRNA. Kaplan–Meier analysis revealed that patients with elevated *EV-associated miR-638* expression had significantly reduced disease-free survival (DFS) compared with those with lower expression levels. The 2-year DFS rate was 47.1% in the high-expression group versus 77.4% in the low-expression group. In contrast, no significant differences in DFS were observed according to the expression levels of *miR-663a*, *miR-3648*, or *miR-4258*. Following hepatectomy, distant metastasis occurred in 5 patients, whereas intrahepatic metastasis occurred in 21 patients. High *EV-associated miR-638* expression was significantly associated with increased frequencies of both distant metastasis (11.1% vs. 7.4%) and intrahepatic metastasis (51.9% vs. 25.9%), compared with the low-expression group. No significant associations were found between the expression levels of *miR-663a*, *miR-3648*, or *miR-4258* and metastatic incidence. Overall survival did not differ significantly according to the expression levels of any of the examined miRNAs [67].

Circulating *EV-associated miR-21* and long non-coding RNA activated by transforming growth factor-β (*lncRNA-ATB*) were significantly associated with TNM stage and other adverse prognostic features, including portal vein thrombosis and advanced T stage. Multivariate Cox regression analysis identified elevated levels of both *miR-21* and *lncRNA-ATB* as independent predictors of mortality and disease progression, together with larger tumor size and increased C-reactive protein levels. Kaplan–Meier survival analysis further demonstrated that patients with higher circulating *EV-associated miR-21* and *lncRNA-ATB* exhibited significantly reduced overall survival and progression-free survival. Collectively, these findings indicate that circulating EV-derived non-coding RNAs, particularly *miR-21* and *lncRNA-ATB*, may serve as promising prognostic biomarkers and potential therapeutic targets in HCC [68].

A study reported that lower *EV-associated miR-122* expression was significantly associated with OS in patients with HCC undergoing transarterial chemoembolization (TACE), highlighting the potential of miRNAs as biomarkers for treatment response monitoring. Furthermore, another study demonstrated that the detection of downregulated plasma *EV-associated miR-192* levels may aid in identifying patients with an unfavorable prognosis who could be considered for adjuvant therapy or for earlier initiation of systemic treatment [69,70].

Previous studies have reported that plasma *EV-associated miR-192* has both diagnostic and prognostic relevance in HCC, with elevated *EV-associated miR-192 levels* significantly associated with reduced overall survival. Zhu et al. also found that elevated circulating *miR-192* levels were correlated with poor OS [71,72].

*miR-16* has been reported to be downregulated in HCC cells, while its restoration suppresses invasion, cell proliferation, and metastasis, supporting its role as a tumor suppressor. In the above-mentioned study by Fründt et al., *EV-associated miR-16* levels were significantly reduced in patients with HCC compared with healthy controls, and lower expression was associated with advanced Barcelona Clinic Liver Cancer (BCLC) stage and metastasis. Moreover, *EV-associated miR-16* demonstrated both diagnostic and prognostic relevance in patients with liver cirrhosis. As shown by Kim et al., the downregulation of miR-16 promoted the progression of liver fibrosis by activating hepatic stellate cells [69,72].

The functional role *of miR-146a* in HCC remains controversial. Recent evidence suggests that *miR-146a* may exert both oncogenic and tumor-suppressive effects by participating in multiple pathways involved in hepatocarcinogenesis. In the study by Fründt et al., *EV-associated miR-146a* levels were increased in patients with HCC but not in those with liver cirrhosis. Multivariate analysis demonstrated that higher *EV-associated miR-146a* expression was significantly associated with a reduced hazard ratio for death, supporting a possible tumor-suppressive role in this context [69,73].

*EV-associated miR-34a* levels were significantly reduced in preoperative HCC patients compared with healthy individuals and postoperative cases. Reduced serum *EV-associated miR-34a* expression was significantly associated with advanced TNM stage, poor tumor differentiation, deeper tumor invasion, vascular invasion, and lymph node metastasis, but showed no significant correlation with standard clinical and biochemical parameters. Multivariate analysis further identified serum *EV-associated miR-34a* as an independent prognostic factor for HCC. These findings support the potential utility of serum *EV-associated miR-34a* as a biomarker for prognosis in HCC [57].

A study published in 2021 included 40 patients diagnosed with HCC. Among them, 20 patients had HCC with lung metastasis, and 32 EV-associated miRNAs were identified as differentially expressed between four patients with HCC with lung metastasis. Among these, 18 miRNAs were significantly upregulated, including *miR-221*, *miR-27a*, *miR-652*, *let-7e*, *miR-140*, *miR-18a*, *miR-361*, *miR-27b*, *miR-6798*, *miR-185*, *miR-20b*, *miR-151b*, *miR-1280*, *miR-1268b*, *miR-4253*, *miR-4454*, *miR-181a*, and *miR-1273h*. In contrast, 5 miRNAs were significantly downregulated (*miR-4720*, *miR-4330*, *miR-8075*, *miR-2277*, and *miR-5189*). ROC analysis demonstrated that *miR-18a*, *miR-20b*, and *miR-27a* could distinguish metastatic HCC from non-metastatic HCC. Furthermore, the prognostic performance of the three-miRNA combination (*miR-18a*, *miR-221*, and *miR-20b*) was superior to that of individual miRNAs. Kaplan–Meier survival analysis showed that elevated expression of *miR-27a*, *miR-652*, *let-7e*, *miR-18a*, *miR-20b*, and *miR-221* was significantly associated with poorer OS, while increased *miR-652* expression was also linked to reduced DFS [74].

In addition, EV-associated miRNAs have emerged as key regulators of therapeutic resistance in HCC by mediating intercellular transfer of drug-resistant phenotypic characteristics within the tumor microenvironment, such as *miR-21*, *miR-221/222*, *miR-210*, *miR-122*, and *miR-181a*, which collectively modulate key resistance-associated pathways such as PI3K/AKT signaling, apoptosis, EMT, and hypoxia adaptation. *EV-associated miR-21* and *miR-221/222* have been consistently linked to sorafenib resistance through inhibition of tumor suppressors such as PTEN and pro-apoptotic factors, while EV-mediated transfer of *miR-210* supports tumor cell survival under hypoxic and therapeutic stress. In contrast, *EV-associated miR-122* has been shown to enhance drug sensitivity by targeting oncogenic signaling pathways. Overall, EV-associated miRNA transfer contributes to intratumoral heterogeneity and adaptive resistance in HCC, representing a potential target for therapeutic intervention. These mechanisms have been comprehensively reviewed in recent studies addressing EV-miRNA-mediated regulation of HCC progression and drug response [75].

The prognostic value of EV-associated miRNAs in hepatocellular carcinoma is summarized in Table 3, highlighting their associations with survival outcomes, disease progression, and therapeutic response.

## 7. Clinical Translation of EV-Associated miRNAs as Liquid Biopsy Biomarkers Standardization and Current Clinical Evidence

As previously discussed, EVs exert a complex and dynamic influence on the development of HCC, functioning as critical mediators of communication between malignant cells and the surrounding tumor microenvironment. EV-associated miRNAs contribute to several hallmarks of cancer, including neovascularization, metastatic dissemination, immune modulation, and metabolic reprogramming in hepatic tissue [76]. EVs secreted by HCC cells transport oncogenic proteins and diverse nucleic acids capable of inducing phenotypic alterations in non-malignant cells, thereby supporting uncontrolled proliferation and maintenance of tumor growth. These processes highlight the direct involvement of EVs in the initiation and progression of HCC [77]. EVs also hold considerable promise as minimally invasive biomarkers for the early identification of HCC, as they are detectable in blood and urine and carry molecular cargo reflective of their originating tumor cells. Their protein and miRNA content are specifically linked to HCC, enabling detection potentially prior to the onset of overt clinical manifestations. In parallel, distinct EV-associated miRNA expression signatures have been shown to differentiate HCC from benign liver conditions and healthy individuals, supporting their utility as non-invasive diagnostic tools that may complement conventional imaging techniques and tissue biopsy [78].

In addition, HCC-derived EVs can condition distant sites for metastatic colonization by remodeling the extracellular matrix and promoting the formation of a pre-metastatic niche. This process is mediated through the transfer of metastasis-associated proteins and regulatory miRNAs and is implicated in key tumor-related mechanisms, including angiogenesis, metastasis, drug resistance, immune modulation, and epithelial–mesenchymal transition (EMT) [79].

The application of EVs in HCC management represents a promising advancement in oncology, particularly due to their potential in non-invasive diagnostics and the development of novel therapeutic strategies. Because EVs reflect the molecular characteristics of their parent tumor cells, they provide insight into tumor biology and may support more personalized treatment approaches. The integration of EV-based technologies, especially EV-associated miRNA, into clinical practice could improve disease monitoring and therapeutic decision-making in HCC. Nevertheless, further investigation is required to validate their clinical utility and to facilitate translation into routine medical care [76].

Despite the growing body of evidence supporting the clinical relevance of EV-associated miRNAs in HCC, several aspects related to their translational implementation remain under active investigation. Multiple EV isolation and characterization approaches, including ultracentrifugation, size-exclusion chromatography, and commercial precipitation-based methods, are currently used across studies. However, methodological variability involving sample type, pre-analytical processing, RNA extraction, and miRNA detection platforms may influence analytical outcomes and complicate cross-study comparisons. In this context, the establishment of standardized workflows and reproducible analytical protocols is essential for improving data consistency and facilitating future clinical integration. Recent efforts led by the International Society for Extracellular Vesicles (ISEV) represent important steps toward harmonizing EV research and supporting the development of clinically applicable EV-based biomarkers in HCC [80].

## 8. Challenges, Limitations, and Future Perspectives

Although EV-associated miRNAs represent promising biomarkers for hepatocellular carcinoma, several important limitations continue to hinder their routine clinical implementation. One major challenge is the limited reproducibility of currently proposed biomarkers, as many findings have not yet been consistently validated across independent cohorts or multicenter studies. In addition, substantial biological heterogeneity exists among EV populations due to their diverse cellular origins and dynamic molecular cargo, potentially affecting biomarker reliability and interpretation. Technical issues, including the co-isolation of non-EV contaminants and the absence of universally accepted quantification methods, further complicate downstream analyses and clinical standardization. Another important limitation relates to the relatively small sample sizes and inter-individual variability observed in many published studies, which may contribute to inconsistent diagnostic and prognostic results. Future research should therefore focus on large-scale prospective validation studies, harmonized analytical approaches, and the identification of highly reproducible EV-associated miRNA signatures across different HCC stages and etiologies. Addressing these methodological and cohort-related factors will be critical to establishing serum EV-associated miRNAs as robust, accurate, and clinically applicable biomarkers for early detection and prognostic assessment in HCC [80].

## 9. Conclusions

Exosomal microRNAs represent a promising translational tool in hepatocellular carcinoma, bridging molecular tumor biology with clinical application. Their stability in circulation and ability to reflect the genetic and functional profile of tumor cells make them attractive candidates for non-invasive liquid biopsy approaches. Growing evidence supports their value in early detection, risk stratification, therapeutic monitoring, and prognostic assessment, often demonstrating performance comparable to or exceeding that of conventional biomarkers.

Despite current challenges related to methodological standardization and large-scale validation, ongoing advances in EV isolation and molecular profiling are accelerating their clinical feasibility. Future large-scale studies and standardized analytical approaches are required to validate their clinical utility and enable translation into routine practice.

## Figures and Tables

**Table 1:** EV-associated miRNAs as diagnostic biomarkers in hepatocellular carcinoma (HCC).

Reference	EV-Associated miRNA(s)	EV Source	Expression in HCC	Clinical Relevance	Key Findings
Li et al., 2024 [50]	*let-7a*, *miR-150*, *miR-21*, *miR-200a*, *miR-125a*	Fucosylated EVs (serum)	Differentially expressed (panel)	Diagnosis, early detection	miRNA panel showed high diagnostic accuracy; superior to AFP/DCP
Cho et al., 2020 [51]	*miR-25-3p*, *miR-4661-5p*, *miR-1269a*, *miR-4746-5p*	Serum EVs	Upregulated	Diagnosis, prognosis, early detection	miR-4661-5p—high diagnostic performance; superior to AFP
Yang et al., 2022 [52]	*miR-26a*, *miR-29c*, *miR-199a*	Serum EVs	Downregulated	Diagnosis	High diagnostic accuracy; superior to plasma miRNAs and AFP
Lin et al., 2019 [53]	*miR-26a*, *miR-21* and *miR-29c*	Serum EVs	Downregulated	Diagnosis	Reduced expression in HCC compared with cirrhotic and HBV groups
Jang et al., 2022 [54]	*miR-720*	Serum EVs	Upregulated	Diagnosis, early detection	High diagnostic accuracy; superior to AFP and PIVKA-II
Sun et al., 2021 [55]	*miR-101*, *miR-125b*	Serum EVs	Downregulated	Diagnosis	Combined analysis improved diagnostic accuracy
Cho et al., 2020 [56]	*miR-10b-5p*	Serum EVs	Upregulated	Early detection	High diagnostic performance in early-stage HCC
Chen et al., 2022 [57]	*miR-34a*	Serum EVs	Downregulated	Diagnosis	Improved diagnostic performance when combined with AFP
Jingwen et al., 2026 [58]	*miR-27a-3p*, *miR-493-3p*	Serum EVs	Differentially expressed	Diagnosis	Improved diagnostic accuracy when combined with AFP
Wang et al., 2020 [59]	*miR-122*, *miR-96*, *miR-21*	Serum EVs	Upregulated	Diagnosis	High discriminative ability for HCC diagnosis
Sohn et al., 2015 [60]	*miR-18a*, *miR-221*, *miR-222*, *miR-224 ↑*; *miR-101*, *miR-106b*, *miR-122*, and *miR-195 ↓*	Serum EVs	Up/downregulated	Diagnosis	Elevated miR-221/222 levels; strong discriminatory potential
Molina-Pelayo et al., 2025 [61]	*miR-148b-3p*, *miR-19a-3p*, *miR-183-5p*, *miR-34a-5p*, *miR-215-5p*	Plasma EVs	Upregulated	Diagnosis	High diagnostic performance across HCC stages
Qi et al., 2022 [62]	*miR-34a*, *miR-192*, *miR-122*, *miR-200a*	Circulating EVs	Differentially expressed	Fibrosis/risk stratification	Strong correlation with fibrosis
Muhammad Yusuf et al., 2020 [63]	*miR-182*, *miR-373*, *miR-301a*	Serum/ascitic EVs	Upregulated	NASH-related HCC	Potential biomarkers for NASH-related HCC

The arrows indicate the levels of miRNAs detected in serum-derived EVs, showing whether they are increased or decreased.

**Table 2:** Diagnostic performance of AFP and EV-associated miRNAs (miR-19-3p, miR-30d-5p, miR-16-5p, miR-451a, and miR-223-3p) in the detection of NBNC-HCC.

	AFP	miR-19-3p	Mir-19-3p and AFP	miR-30d-5p	miR-16-5p	miR-451a	miR-223-3p
**Cut-off**	20 ng/mL	1.9	1.9 and 20 ng/mL	1.0	0.8	1.0	1.5
**Sensitivity (%)**	44.3	74.3	89.9	71.4	68.6	65.7	62.9
**Specificity (%)**	97.1	79.0	76.7	66.7	70.5	71.4	63.8

Adapted to [64].

**Table 3:** Prognostic value of EV-associated miRNAs in hepatocellular carcinoma.

Reference	EV-Associated miRNA(s)	EV Source	Expression in HCC	Prognostic Value	Key Findings
Liu et al., 2017 [65]	*miR-125b*	Serum EVs	Downregulated	Recurrence, OS	Associated with recurrence and OS; improved prognostic accuracy with MVI
Yang et al., 2020 [66]	*miR-92a-3p*	Plasma EVs	Upregulated	Metastasis	Associated with metastasis and EMT progression
Cho et al., 2020 [56]	*miR-215-5p*	Serum EVs	Upregulated	DFS, tumor progression	Associated with shorter DFS, advanced stage, and vascular invasion
Yokota et al., 2021 [67]	*miR-638*	Serum EVs	Upregulated	DFS, metastasis	Associated with reduced DFS and increased metastasis
Lee et al., 2019 [68]	*miR-21*	Circulating EVs	Upregulated	OS, PFS	Associated with poor OS and disease progression
Fründt et al., 2021 [69]; Suheiro et al., 2018 [70]	*miR-122*, *miR-192*	Plasma/serum EVs	Downregulated	OS, treatment response	Associated with OS and treatment response
Kim et al., 2018 [72]	*miR-16*	Serum EVs	Downregulated	Progression, metastasis	Associated with advanced stage and metastasis
Wang et al., 2019 [73]	*miR-146a*	Serum EVs	Upregulated	OS	Associated with reduced mortality risk
Chen et al., 2022 [57]	*miR-34a*	Serum EVs	Downregulated	OS, aggressiveness	Associated with advanced stage and tumor invasion
Huang et al., 2021 [74]	*miR-18a*, *miR-20b*, *miR-221*, *miR-27a*, *miR-652*, *let-7e*	Plasma EVs	Upregulated	OS, DFS, metastasis	Associated with poor OS and DFS; improved prognostic prediction

## Data Availability

All data associated with this paper are contained within the article.
